# Short-term changes in CRS-R scores during transcutaneous auricular vagus nerve stimulation in prolonged disorders of consciousness: a single-arm exploratory study

**DOI:** 10.3389/fneur.2026.1815827

**Published:** 2026-09-16

**Authors:** Weihang Zhai, Meng Li, Guowei Lü, Yi Yang, Fang Li, Beibei Yin, Long Xu, Jinling Zhang, Chen Xin, Guohairong Pan, Jingwei Kong, Xiaoli Geng, Xueling Chen, Yifei Wang, Jianghong He, Peijing Rong

**Affiliations:** 1 Institute of Basic Research in Clinical Medicine, China Academy of Chinese Medical Sciences, Beijing, China; 2 Department of Trauma Surgery, Zhengzhou Central Hospital Affiliated to Zhengzhou University, Zhengzhou, China; 3 Department of Neurosurgery, Beijing Tiantan Hospital, Capital Medical University, Beijing, China; 4 Institute of Acupuncture and Moxibustion, China Academy of Chinese Medical Sciences, Beijing, China

**Keywords:** CRS-R, minimally conscious state, prolonged disorders of consciousness, transcutaneous auricular vagus nerve stimulation, vegetative state

## Abstract

**Objectives:**

To describe short-term behavioral changes observed during a 2-week transcutaneous auricular vagus nerve stimulation (taVNS) intervention in patients with prolonged disorders of consciousness (pDOC) and to exploratorily compare these changes across baseline diagnostic states.

**Methods:**

This single-center, single-arm, open-label exploratory study included 50 patients with pDOC (25 with VS/UWS, 16 with MCS−, and 9 with MCS+) who received taVNS in addition to standard care for 2 weeks. CRS-R assessments were conducted at baseline, week 1, and week 2. The primary outcome was the longitudinal change in the CRS-R total score across the three assessment time points.

**Results:**

At week 2, the mean change in CRS-R total score from baseline was 2.40 ± 2.08 points (95% CI, 1.81–2.99), with no significant difference among the diagnostic groups. Upward diagnostic transitions occurred in 9/25 (36.0%) patients with VS/UWS (VS → MCS−, *n* = 7; VS → MCS+, *n* = 2) and Six of 16 (37.5%) patients with MCS− transitioned to MCS+, but in none of the patients with MCS+.

**Conclusion:**

Short-term CRS-R changes and diagnostic transitions were observed during the 2-week taVNS intervention. Because the study lacked a control group and used unblinded outcome assessment, these changes cannot be attributed specifically to taVNS. The subgroup and early-outcome analyses are exploratory and require confirmation in adequately powered, sham-controlled studies with blinded assessment and longer follow-up.

## 1 Introduction

Prolonged disorders of consciousness (pDOC) refer to a state of loss of consciousness caused by various severe brain injuries, lasting for more than 28 days (1, 2). This state typically includes vegetative state (VS)/unresponsive wakefulness syndrome (UWS) and minimally conscious state (MCS). VS/UWS is a condition in which basic brainstem reflexes and sleep–wake cycles are preserved. Patients can open their eyes spontaneously or in response to stimulation but lack conscious reactions (3). In contrast, MCS is characterized by discontinuous and fluctuating clear signs of consciousness in patients after severe brain injury (4). Further subdivided, MCS can be categorized into minimally conscious state minus (MCS-) and minimally conscious state plus (MCS+). MCS- is characterized by lower-level purposeful behaviors, such as visual pursuit or localization to noxious stimulation, without language-dependent responses (5, 6). Nationwide prevalence data also show substantial behavioral heterogeneity among institutionalized patients diagnosed with MCS (7). MCS+ is defined by the presence of at least one language-dependent behavior (command-following, intelligible verbalization, or intentional communication). Preserved language-related behaviors are associated with better functional recovery, whereas functional communication or functional object use marks emergence from MCS (8, 9).

Non-invasive neuromodulation has become an active area of pDOC research, with studies evaluating several modalities and patient subgroups (10–15). taVNS stimulates auricular vagal afferents in the concha without an implanted device, offering a comparatively simple approach without perioperative implantation risks (16).

The review article by our team summarizes the clinical research progress and challenges of taVNS in the treatment of pDOC in recent years (16). Our team previously reported a case of taVNS treatment in a patient with pDOC (17). However, this study could not rule out the possibility that the patient’s recovery was spontaneous. Subsequent research revealed that the preservation of auditory function may be one of the key conditions for taVNS to take effect in treating pDOC (18). Another study by our team showed that after taVNS treatment, MCS patients exhibited decreased delta band energy and increased beta band energy in the electroencephalography (EEG) power spectrum. In contrast, VS patients showed the opposite pattern (19). Jakob Hakon and his colleagues administered taVNS to 5 pDOC patients for 8 weeks. The results showed that 3 out of the 5 patients experienced consciousness improvement. Specifically, 2 patients improved from VS and MCS to emergence from minimally conscious state (EMCS), while 1 patient improved from VS to MCS (20). In another study, 14 pDOC patients received taVNS treatment on the left ear for 4 weeks. One MCS patient showed new signs of consciousness at the end of the 4-week stimulation period (21). In the aforementioned studies, only one patient reported intermittent ear pruritus during the stimulation process. This finding suggests that taVNS is a feasible and safe therapeutic approach for patients with pDOC.

Compared with other neuromodulation strategies investigated for disorders of consciousness, such as rTMS and tDCS, taVNS has several practical advantages, including peripheral non-invasive delivery, relative ease of bedside implementation, and lower procedural burden. At the same time, the evidence base for taVNS in pDOC remains limited, and its place within the current therapeutic landscape should be considered exploratory.

This study aimed to describe short-term behavioral changes observed during a 2-week taVNS intervention in patients with pDOC and to exploratorily examine whether these changes differed across baseline diagnostic states.

## 2 Materials and methods

### 2.1 Study participants

From December 2024 to October 2025, a total of 50 patients with pDOC were enrolled from the Department of Neurosurgery at Beijing Tiantan Hospital, Capital Medical University, including 25 patients with VS, 16 with MCS-, and 9 with MCS+.

For this exploratory cohort, the age range was set at 18–80 years to restrict enrollment to adults while retaining clinically stable older patients seen in routine practice, because advanced age may reduce, but does not eliminate, recovery potential. The inclusion and exclusion criteria were as follows.

Inclusion criteria: (1) diagnosis of pDOC; (2) age between 18 and 80 years; (3) disease duration ≥1 month.

Exclusion criteria: (1) neurodegenerative diseases, postoperative coma due to intracranial infection, or brain tumors; (2) severe systemic complications; (3) participation in other clinical trials within the previous 3 months; (4) a history of severe allergy or allergic constitution.

### 2.2 Stimulation protocol

The stimulation site of taVNS (SDZ-IIB, Suzhou Medical Supplies Factory) is the concha area of the ear, mainly the cymba conchae and concha cavity. A pair of identical-looking clips are placed on both ears. The clips are designed with three carbon-impregnated silicone tips, one of which serves as the common terminal to support the posterior surface of the auricle, and the other two tips are designed to stimulate two skin surface points, one on the outer ear and the other on the cymba concha. The two silicone tips at the outer ear point are, respectively, placed in the concha cavity and cymba conchae, as shown in Figure 1A.

**Figure 1:**
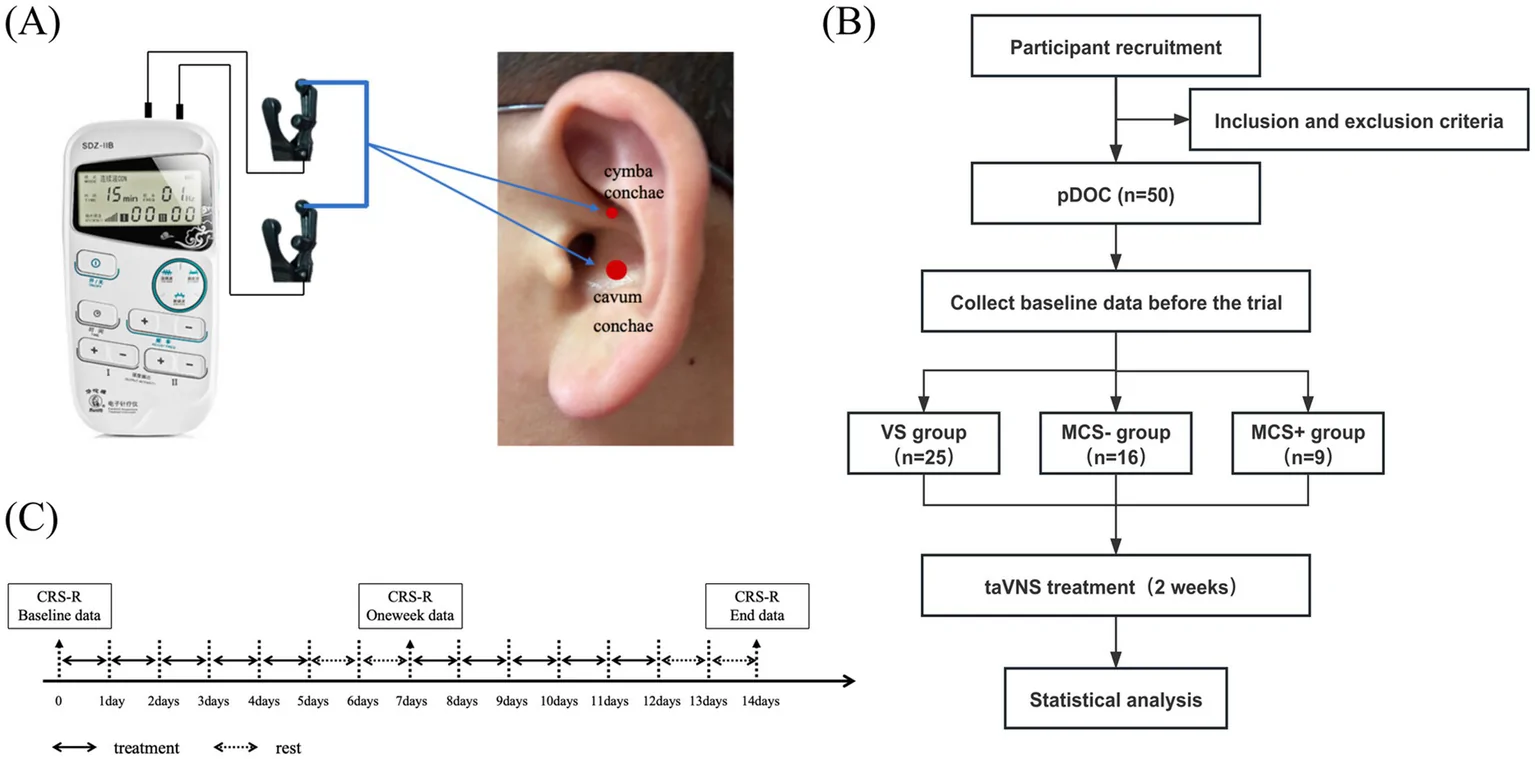
**(A)** Schematic diagram of transcutaneous auricular vagus nerve stimulation. **(B)** Flowchart of the study. **(C)** Flowchart of the treatment and data acquisition procedures.

In this study, all patients received a 2-week taVNS intervention in addition to standard care. Stimulation was delivered to the auricular “Heart” and “Kidney” acupoints using a dense–disperse waveform at a frequency of 4/20 Hz. The stimulation intensity was adjusted to avoid significant heart rate fluctuations, with a current amplitude of approximately 1–1.5 mA. Each treatment session lasted 30 min and was administered twice daily (once in the morning and once in the evening), following a regimen of 5 consecutive treatment days followed by 2 days of rest, for a total duration of 2 weeks. CRS-R assessments were conducted at three time points: before treatment, at the first week of treatment, and after treatment. The overall trial flow is illustrated in Figure 1B, and the treatment flowchart for each patient is shown in Figure 1C.

### 2.3 CRS-R

The CRS-R is a widely used standardized behavioral instrument for detecting signs of consciousness and differentiating VS/UWS from MCS (22–27). Its six subscales assess auditory, visual, motor, oromotor/verbal, communication, and arousal functions, with a total score of 23 points. CRS-R assessments were independently performed by two experienced clinicians at baseline, week 1, and week 2. Disagreements were adjudicated by a senior clinician. Assessors were not blinded to assessment time point or treatment phase.

### 2.4 Sample size

At the time of study design, the available evidence on taVNS in patients with prolonged disorders of consciousness consisted mainly of case reports and small pilot or feasibility cohorts, which did not provide a sufficiently reliable effect-size estimate for the present longitudinal and subgroup analyses. We therefore consecutively enrolled all eligible patients during the predefined recruitment period from December 2024 to October 2025, and the final sample size was determined by recruitment feasibility. This study was exploratory rather than confirmatory and was not powered to establish treatment efficacy or to support definitive comparisons among the VS/UWS, MCS-, and MCS+ subgroups. The estimates obtained from this cohort may inform effect-size assumptions and sample-size planning for future adequately powered, sham-controlled trials.

### 2.5 Statistical analysis

Statistical analyses were performed using SPSS version 26.0. Continuous variables were assessed for normality and are presented as mean ± standard deviation (SD) when normally distributed or as median (interquartile range, IQR) otherwise. Baseline continuous variables were compared among the three diagnostic groups using one-way analysis of variance (ANOVA) or the Kruskal-Wallis test. Categorical variables are presented as *n* (%) and were compared using the chi-square test or Fisher’s exact test, as appropriate.

CRS-R total and subscale scores measured at baseline, week 1, and post-treatment were analyzed using a two-way mixed-design repeated-measures ANOVA, with time as the within-subject factor and baseline diagnostic group (VS/UWS, MCS-, and MCS+) as the between-subject factor. The main effects of time and group and the time-by-group interaction were examined. Sphericity was assessed using Mauchly’s test, and the Greenhouse–Geisser correction was applied when the assumption of sphericity was violated. Where appropriate, pairwise comparisons of estimated marginal means between assessment time points within each diagnostic group were performed with Bonferroni adjustment. Bonferroni-adjusted *p* values were used for the significance annotations shown in the relevant figure panels. When the assumptions of repeated-measures ANOVA were not adequately met, within-group changes over time were assessed using the Friedman test, followed, where appropriate, by Bonferroni-adjusted Wilcoxon signed-rank tests.

Three CRS-R total-score changes were calculated: t_x_ = week1—baseline, t_y_ = post-treatment—week 1, and t_z_ = post-treatment—baseline. For each change score, differences among the VS/UWS, MCS-, and MCS+ groups were evaluated using one-way ANOVA or the Kruskal-Wallis test, as appropriate. These change-score analyses were used only for the between-group comparisons presented in the Results. Changes in CRS-R scores are reported as mean differences with SDs and 95% confidence intervals (CIs). All statistical tests were two-sided. Bonferroni-adjusted *p* values were reported for multiple pairwise comparisons, and an adjusted *p* < 0.05 was considered statistically significant.

## 3 Results

### 3.1 Baseline demographic and clinical characteristics of the three groups of patients

A total of 50 patients with pDOC were enrolled in this study, including 25 VS, 16 MCS-, and 9 MCS+ patients. There were no significant differences among the three groups in age, sex, etiology, or disease duration (*p* > 0.05), as shown in Table 1.

**Table 1:** Clinical baseline data of the three groups of patients.

Variables	VS group (*n* = 25)	MCS-group (*n* = 16)	MCS+group (*n* = 9)	*p*-value
Gender				
Male	21	12	4	0.091^b^
Female	4	4	5	
Age	55.76 ± 12.01	56.81 ± 12.46	59.22 ± 15.18	0.784^a^
Etiology				0.230^b^
TBI	2	4	2	
ICH	23	12	7	
Post-injure (month)	3.19 ± 3.67	3.06 ± 3.53	2.39 ± 1.95	0.592^c^

Gender (F and M); Etiology (TBI and ICH). CRS-R, coma recovery scale-revised; F, female; M, male; MCS-, minimally conscious state minus; MCS+, minimally conscious state plus; ICH, intracerebral hemorrhage; TBI, traumatic brain injuries; VS, vegetative state/Unresponsive Wakefulness Syndrome.

a One-way analysis of variance (ANOVA).

b Fisher’s exact test.

c Kruskal–Wallis test.

### 3.2 Improvement rate of consciousness state and changes in CRS-R scores after short-term taVNS

#### 3.2.1 Improvement in consciousness state among the three groups of patients

A total of 15 patients showed an improvement in consciousness state after treatment, yielding an overall improvement rate of 30%. Specifically, 7 patients improved from VS to MCS-, 2 patients from VS to MCS+, and 6 patients from MCS- to MCS+ . The improvement rates were 36% in the VS group, 37.5% in the MCS- group, while no patients in the MCS+ group exhibited an improvement in consciousness state. The improvement outcomes of the three groups are illustrated in Figure 2.

**Figure 2:**
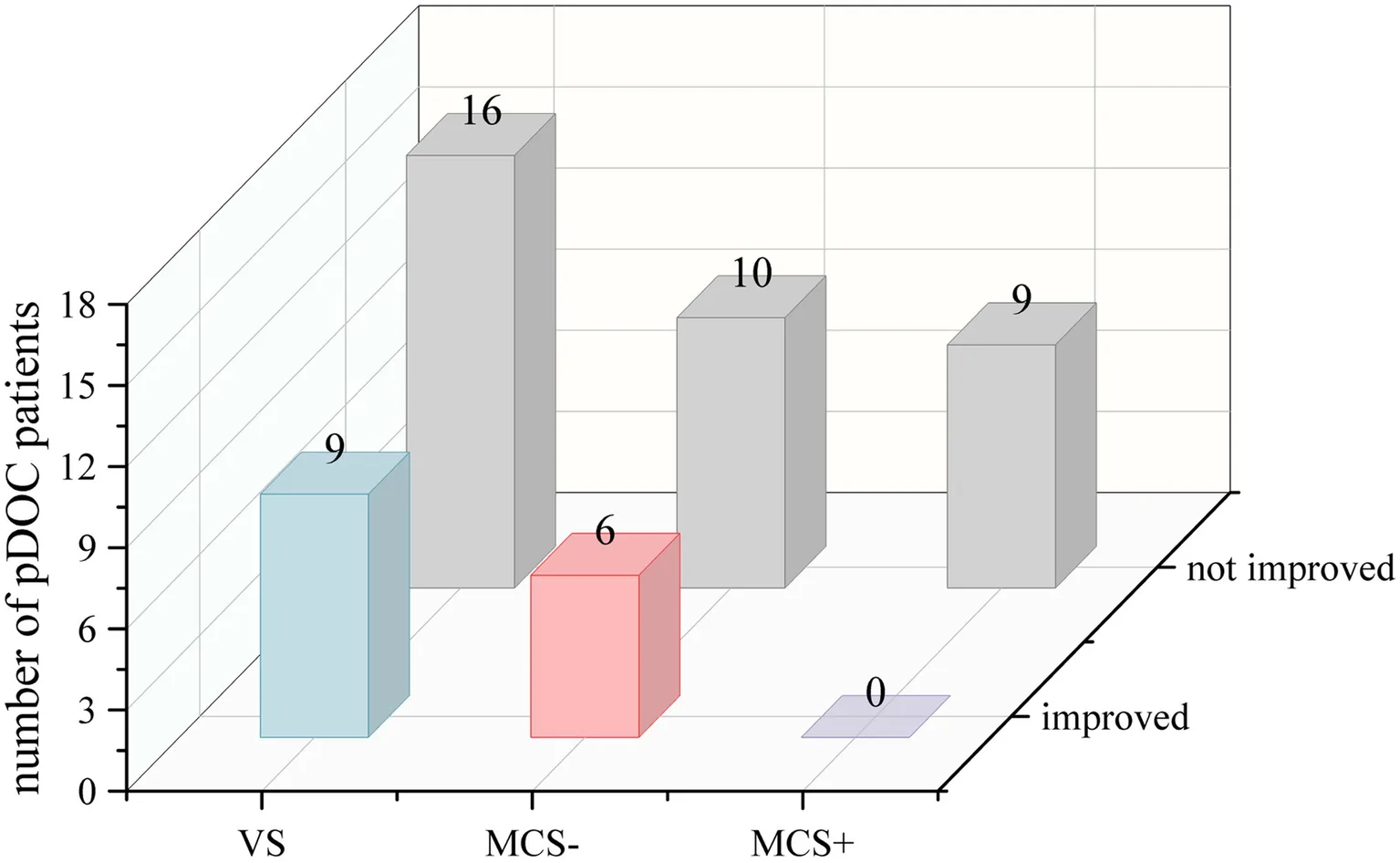
Numbers of patients with improved and not-improved consciousness status after short-term taVNS treatment, by baseline diagnostic group.

#### 3.2.2 Changes in CRS-R scores at different time points among the three groups of patients

After short-term taVNS, the mean CRS-R total scores increased from 6.64 ± 3.43 at baseline to 8.04 ± 3.74 at week 1 and 9.04 ± 3.99 post-treatment, corresponding to a mean baseline-to-post-treatment change of 2.40 ± 2.08 points (95% CI, 1.81–2.99) across all three groups. In the VS group, significant differences were observed between baseline and week 1, between week 1 and post-treatment, and between baseline and post-treatment (*p* < 0.01, *p* < 0.05, *p* < 0.01). In the MCS- group, significant differences were found between baseline and week 1, as well as between baseline and post-treatment (*p* < 0.05, *p* < 0.05). In the MCS+ group, a significant difference was observed between baseline and post-treatment (*p* < 0.05), as shown in Figure 3A.

**Figure 3:**
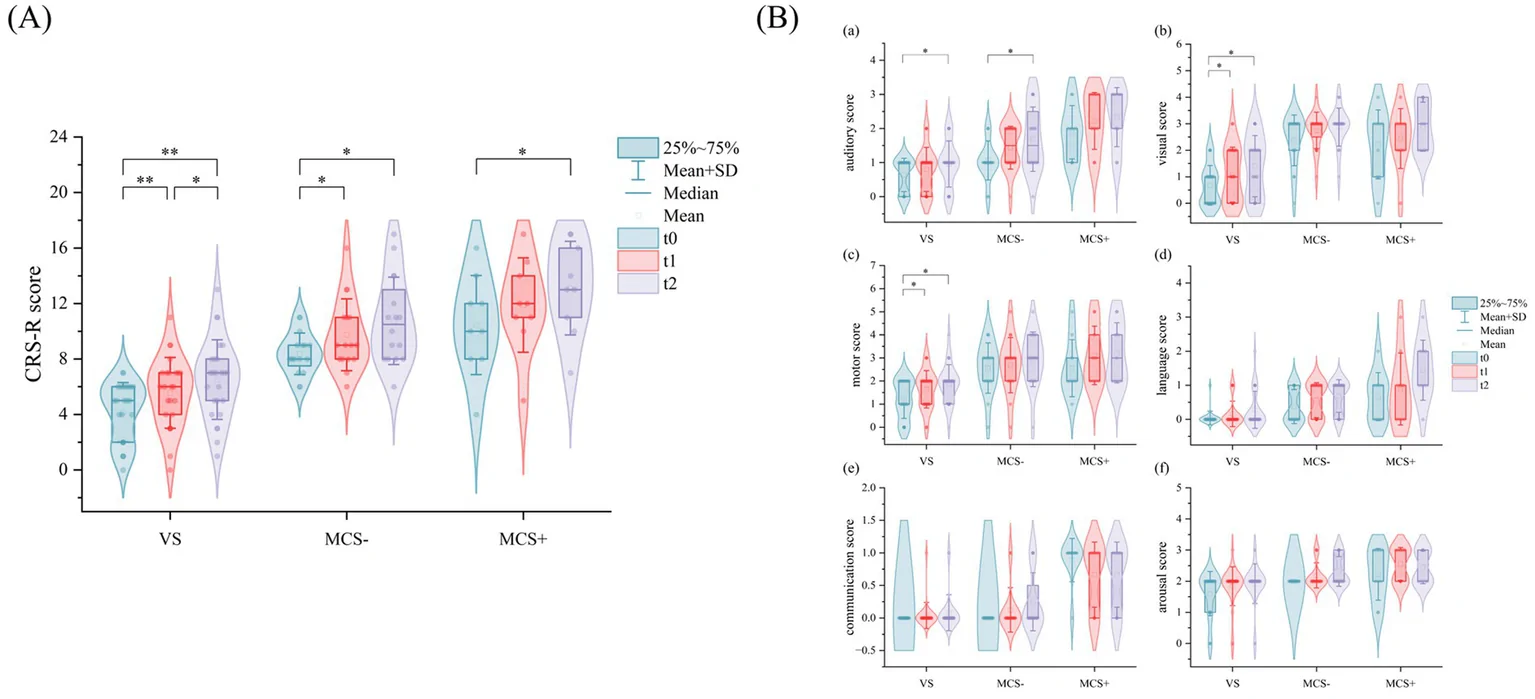
Changes in CRS-R scores over time. **(A)** Changes in total CRS-R scores over time. T0, baseline; T1, week 1 of treatment; T2, post-treatment (*p < 0.05, **p < 0.01). **(B)** Changes in CRS-R subscale scores over time. (a) Auditory score; (b) Visual score; (c) Motor score; (d) Oromotor/verbal score; (e) Communication score; and (f) Arousal score. T0, baseline; T1, week 1 of treatment; T2, post-treatment (*p < 0.05, **p < 0.01).

In the VS group, the auditory subscale score showed a statistically significant difference between post-treatment and baseline (*p* < 0.05). The visual subscale score exhibited significant differences between baseline and week 1, as well as between baseline and post-treatment (*p* < 0.05, *p* < 0.05). The motor subscale score also demonstrated significant differences between baseline and week 1, and between baseline and post-treatment (*p* < 0.05, *p* < 0.05). In the MCS- group, the auditory subscale score showed a statistically significant difference between post-treatment and baseline (*p* < 0.05), as shown in Figure 3B. These subgroup-specific changes should be interpreted cautiously as exploratory findings, given the limited sample sizes within each subgroup.

#### 3.2.3 Comparison of CRS-R score increments across different treatment stages

We defined the difference in CRS-R scores between week 1 and baseline as t*_x_*, the difference between post-treatment and week 1 as t*_y_*, and the difference between post-treatment and baseline as t*_z_*. Between-group and within-group comparisons of t*_x_*, t*_y_*, and t*_z_* were performed among the three groups. Mean baseline-to-post-treatment changes were 2.36 ± 2.00, 2.38 ± 2.47, and 2.56 ± 1.74 points in the VS/UWS, MCS-, and MCS+ groups, respectively, with no significant between-group difference (*p* > 0.05), as shown in Figure 4.

**Figure 4:**
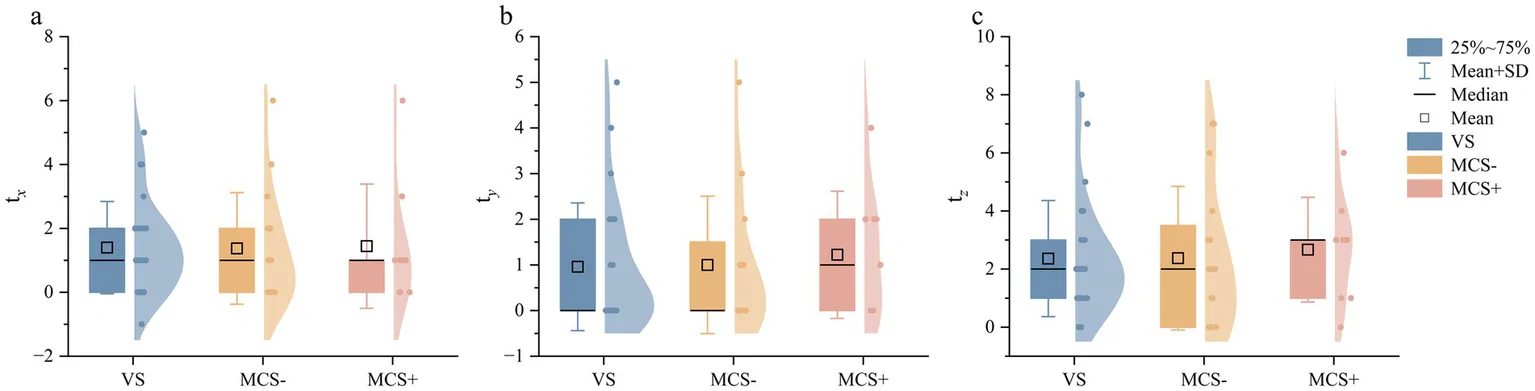
Comparison of CRS-R total-score increments among the three baseline diagnostic groups. **(a)** t_x = week 1 − baseline; **(b)** t_y = post-treatment − week 1; and **(c)** t_z = post-treatment − baseline.

## 4 Discussion

This single-center, single-arm exploratory study characterized short-term behavioral changes in 50 patients with prolonged disorders of consciousness during a 2-week period in which taVNS was administered in addition to standard care. By week 2, 15 patients (30%) showed an upward diagnostic transition: 9 of 25 patients with VS/UWS and 6 of 16 patients with MCS-, whereas no upward transition was observed among the 9 patients with MCS+. The mean CRS-R total score increased by 2.40 points from baseline to post-treatment (95% CI, 1.81–2.99), with comparable changes across the three diagnostic groups. Exploratory subscale analyses suggested changes mainly in auditory, visual, and motor functions. However, no statistically significant between-group differences in the magnitude of CRS-R change were detected. The present results therefore describe temporal behavioral patterns observed during the intervention period rather than differential or causal effects of taVNS across diagnostic subgroups.

These observations are broadly consistent with prior case and cohort reports of behavioral improvement during taVNS (17, 20, 21) and with a sham-controlled trial in which between-group CRS-R differences emerged only after 3–4 weeks (28). Nevertheless, the early temporal changes and subscale patterns observed in the present study should be interpreted only as hypothesis-generating findings. Behavioral responsiveness in pDOC is intrinsically variable, and repeated standardized examinations can increase the probability of detecting intermittent or subtle signs of consciousness (29, 30). Consequently, the observed score changes may reflect a combination of natural clinical fluctuation, improved detection through repeated assessment, concurrent standard care, and a possible intervention-related contribution. The subscale findings do not establish that taVNS preferentially affects specific sensory or motor domains.

More detailed comparison with earlier taVNS studies highlights both concordant observations and important differences. The first case report described a transition from VS/UWS to MCS after 4 weeks of bilateral taVNS (17). An 8-week feasibility study of five patients also reported CRS-R gains in three participants (20). In a 14-patient chronic cohort, CRS-R improvement occurred in five of eight MCS patients but in none of six VS/UWS patients. Only one improvement was present at treatment completion, whereas four additional changes appeared during the subsequent 4-week follow-up (21). A sham-controlled trial in MCS likewise found no active-sham CRS-R difference after 1 or 2 weeks, with differences emerging after 3–4 weeks (28, 29).

Several neurophysiological pathways could provide a plausible framework for these behavioral observations. Stimulation of the cymba conchae may recruit auricular vagal afferents that project to the nucleus tractus solitarius and influence the locus coeruleus and related arousal nuclei (31, 32). Through noradrenergic and related neuromodulatory pathways, this ascending input may alter thalamocortical signaling, cortical excitability, and communication within salience and frontoparietal networks. In pDOC, a small ASL-fMRI study associated preserved auditory responsiveness with broader cerebral blood-flow changes after taVNS (18). An EEG pilot also reported diagnosis-dependent changes in delta and beta activity and functional connectivity, with broader changes among MCS than VS/UWS patients (19). Acute heart-rate variability responses have additionally been proposed as candidate indicators of autonomic engagement and subsequent outcome (33). Together, these findings may help explain why changes in the present study appeared across auditory, visual, and motor CRS-R domains. However, they are more compatible with generalized modulation of arousal and response availability than with selective restoration of individual sensory or motor systems. Clinically, this framework supports serial behavioral assessment alongside objective measures when evaluating early changes. Because no physiological measures were collected, these mechanisms remain hypotheses and cannot be linked causally to the behavioral changes observed here.

The principal limitation of this study is the absence of a sham-stimulation or untreated control group. All participants received taVNS together with standard care; therefore, the observed pre-post changes cannot be separated from spontaneous recovery or natural fluctuations in consciousness. Long-term studies show that recovery can continue well beyond the acute period (34, 35). The observed changes may also reflect changes in concomitant medical or rehabilitation interventions, repeated testing or familiarity effects, increased opportunities to detect inconsistent behaviors, or regression to the mean. Accordingly, neither the increases in CRS-R scores nor the upward diagnostic transitions can be attributed specifically to taVNS. A randomized sham-controlled design with standardized and carefully documented concomitant care is required to determine whether taVNS produces an effect beyond the natural course of recovery and repeated clinical observation.

A second important limitation is that outcome assessment was not blinded to the assessment time point or treatment phase. Although CRS-R examinations were independently performed by two experienced clinicians and disagreements were adjudicated by a senior clinician, these procedures do not eliminate the possibility of expectation-related scoring or detection bias (36, 37). Future trials should use assessors who are blinded to treatment allocation and assessment stage. Whenever feasible, recorded CRS-R examinations should also be evaluated by independent central raters who are unaware of the treatment condition and temporal sequence.

The total sample size was modest and unevenly distributed across the VS/UWS (*n* = 25), MCS- (*n* = 16), and MCS+ (*n* = 9) groups. The particularly small MCS+ subgroup had limited statistical power, and the absence of an upward diagnostic transition or a statistically significant interval change in this subgroup may reflect a Type II error rather than a true absence of responsiveness (38). Similarly, statistical significance within one subgroup and nonsignificance within another subgroup do not themselves demonstrate a difference between the subgroups (39). Because the between-group change comparisons did not demonstrate clear differences, no definitive conclusions can be drawn regarding different response patterns among VS/UWS, MCS-, and MCS+ patients. The absence of a formal *a priori* power calculation further reinforces the exploratory nature of the subgroup analyses. Larger studies with diagnostic-state stratification are needed to estimate subgroup-specific effects with adequate precision.

The study also included a large number of correlated statistical comparisons involving three diagnostic groups, three assessment time points, six CRS-R subscales, and several change-score analyses. Such extensive testing increases the risk of false-positive findings, particularly when nominal *p* values are close to 0.05 (40, 41). Multiplicity adjustment reduces, but does not completely eliminate, this concern in a small exploratory dataset with correlated outcomes. Therefore, interpretation should be based on multiplicity-adjusted results, and all subgroup, subscale, and interval-specific analyses should remain explicitly exploratory. Future confirmatory studies should prespecify a single primary endpoint, limit the number of secondary hypotheses, and use an appropriate hierarchical or multiplicity-control strategy.

Another major limitation is the short intervention and observation period. The assessments ended at the completion of the 2-week intervention, and no post-intervention follow-up was conducted. Long-term TBI cohorts show that recovery trajectories can extend over years (42, 43), so a 2-week observation window cannot establish whether the observed behavioral changes are durable, transient, or measurement-related. Longer follow-up over subsequent weeks and months is required to determine whether diagnostic transitions persist and whether CRS-R changes translate into meaningful functional outcomes.

Generalizability is further limited by the single-center recruitment from a neurosurgical department in Beijing and by the predominance of intracerebral hemorrhage in the cohort. Of the 50 participants, 42 had intracerebral hemorrhage and only 8 had traumatic brain injury. The results may therefore not apply to patients recruited from other clinical settings or to pDOC populations with different etiological distributions. Prior multicenter and longitudinal prognostic studies have included mixed etiologic cohorts (44, 45), underscoring the need for broader representation. Multicenter recruitment with broader etiological representation and stratification by injury type will be necessary.

Finally, the study relied exclusively on behavioral assessment and did not include objective neurophysiological or neuroimaging biomarkers. Although activation of auricular vagal afferents and NTS-centered ascending pathways provides a plausible biological rationale for taVNS (31, 32), the present data cannot determine whether the observed behavioral changes were accompanied by changes in cortical activity, connectivity, or arousal-related networks. Future studies should integrate repeated CRS-R assessment with EEG, evoked potentials, and functional neuroimaging to detect covert brain activation and characterize neural signatures of consciousness (46, 47), while using taVNS-specific physiological measures to assess target engagement.

This study provides preliminary descriptive estimates of short-term CRS-R trajectories and diagnostic transitions during a period of taVNS plus standard care. It does not establish treatment efficacy, identify a preferentially responsive diagnostic subgroup, or validate an early predictive marker. These findings may nevertheless inform the design of adequately powered, multicenter, randomized sham-controlled trials incorporating blinded assessment, prespecified multiplicity control, longer follow-up, standardized concomitant care, and objective biomarker outcomes.

## 5 Conclusion

In this single-center, single-arm exploratory cohort, CRS-R scores increased and 15 of 50 patients showed an upward diagnostic transition during a 2-week period in which taVNS was administered in addition to standard care. These observations cannot be attributed specifically to taVNS because the study lacked a sham or untreated control group and used unblinded outcome assessment. The small and imbalanced diagnostic subgroups, extensive exploratory testing, absence of post-intervention follow-up, single-center recruitment, etiological imbalance, and lack of objective biomarkers further limit interpretation and generalizability. The absence of an upward transition among the 9 patients with MCS+ should not be interpreted as evidence of a different treatment response. The findings should therefore be regarded as hypothesis-generating and used to inform adequately powered, multicenter, randomized sham-controlled trials with blinded assessment, prespecified multiplicity control, longer follow-up, standardized concomitant care, and integrated neurophysiological or imaging outcomes.

## Data Availability

The raw data supporting the conclusions of this article will be made available by the authors, upon a reasonable request.

## References

[1] Kondziella D Bender A Diserens K van Erp W Estraneo A Formisano R . European academy of neurology guideline on the diagnosis of coma and other disorders of consciousness. Eur J Neurol. (2020) 27:741–56. doi: 10.1111/ene.14151, 32090418

[2] Liuzzi P Magliacano A De Bellis F Mannini A Estraneo A. Predicting outcome of patients with prolonged disorders of consciousness using machine learning models based on medical complexity. Sci Rep. (2022) 12:13471. doi: 10.1038/s41598-022-17561-w, 35931703 PMC9356130

[3] Schnakers C Vanhaudenhuyse A Giacino J Ventura M Boly M Majerus S . Diagnostic accuracy of the vegetative and minimally conscious state: clinical consensus versus standardized neurobehavioral assessment. BMC Neurol. (2009) 9:35. doi: 10.1186/1471-2377-9-35, 19622138 PMC2718857

[4] Giacino JT Ashwal S Childs N Cranford R Jennett B Katz DI . The minimally conscious state: definition and diagnostic criteria. Neurology. (2002) 58:349–53. doi: 10.1212/wnl.58.3.349, 11839831

[5] Bruno M-A Majerus S Boly M Vanhaudenhuyse A Schnakers C Gosseries O . Functional neuroanatomy underlying the clinical subcategorization of minimally conscious state patients. J Neurol. (2012) 259:1087–98. doi: 10.1007/s00415-011-6303-7, 22081100

[6] Bruno M-A Vanhaudenhuyse A Thibaut A Moonen G Laureys S. From unresponsive wakefulness to minimally conscious PLUS and functional locked-in syndromes: recent advances in our understanding of disorders of consciousness. J Neurol. (2011) 258:1373–84. doi: 10.1007/s00415-011-6114-x, 21674197

[7] Overbeek BUH van Erp WS Eilander HJ Koopmans RTCM Lavrijsen JCM. Prevalence of the minimally conscious state among institutionalized patients in the Netherlands: a Nationwide study. Neurology. (2023) 101:e2005–13. doi: 10.1212/WNL.0000000000207820, 37857492 PMC10662977

[8] Thibaut A Bodien YG Laureys S Giacino JT. Minimally conscious state ‘plus’: diagnostic criteria and relation to functional recovery. J Neurol. (2020) 267:1245–54. doi: 10.1007/s00415-019-09628-y, 31773246

[9] Giacino JT Sherer M Christoforou A Maurer-Karattup P Hammond FM Long D . Behavioral recovery and early decision making in patients with prolonged disturbance in consciousness after traumatic brain injury. J Neurotrauma. (2020) 37:357–65. doi: 10.1089/neu.2019.6429, 31502498 PMC6964809

[10] Gao Y Bai J Gu Z Xie Y An W Liu Z . Noninvasive neuromodulation for disorders of consciousness: an updated systematic review and meta-analysis. Crit Care. (2025) 29:269. doi: 10.1186/s13054-025-05429-0, 40611258 PMC12224763

[11] Cain JA Spivak NM Coetzee JP Crone JS Johnson MA Lutkenhoff ES . Ultrasonic thalamic stimulation in chronic disorders of consciousness. Brain Stimul. (2021) 14:301–3. doi: 10.1016/j.brs.2021.01.008, 33465497

[12] Fan J Zhong Y Wang H Aierken N He R. Repetitive transcranial magnetic stimulation improves consciousness in some patients with disorders of consciousness. Clin Rehabil. (2022) 36:916–25. doi: 10.1177/02692155221089455, 35322709

[13] Yu J Wu Y Wu B Xu C Cai J Wen X . Sleep patterns correlates with the efficacy of tDCS on post-stroke patients with prolonged disorders of consciousness. J Transl Med. (2022) 20:601. doi: 10.1186/s12967-022-03710-2, 36522680 PMC9756665

[14] Zhang X-H Han P Zeng Y-Y Wang Y-L Lv H-L. The clinical effect of repetitive transcranial magnetic stimulation on the disturbance of consciousness in patients in a vegetative state. Front Neurosci. (2021) 15:647517. doi: 10.3389/fnins.2021.647517, 33994925 PMC8119637

[15] Chen J-M Chen QF Wang ZY Chen YJ Zhang NN Xu JW . Influence of high-frequency repetitive transcranial magnetic stimulation on neurobehavioral and electrophysiology in patients with disorders of consciousness. Neural Plast. (2022) 2022:7195699. doi: 10.1155/2022/7195699, 36437902 PMC9699789

[16] Wang Y Zhang J Zhai W Wang Y Li S Yang Y . Current status and prospect of transcutaneous auricular vagus nerve stimulation for disorders of consciousness. Front Neurosci. (2024) 17:1274432. doi: 10.3389/fnins.2023.1274432, 38260020 PMC10800843

[17] Yu Y-T Yang Y Wang LB Fang JL Chen YY He JH . Transcutaneous auricular vagus nerve stimulation in disorders of consciousness monitored by fMRI: the first case report. Brain Stimul. (2017) 10:328–30. doi: 10.1016/j.brs.2016.12.004, 28017322

[18] Yu Y Yang Y Gan S Guo S Fang J Wang S . Cerebral hemodynamic correlates of transcutaneous auricular vagal nerve stimulation in consciousness restoration: An open-label pilot study. Front Neurol. (2021) 12:684791. doi: 10.3389/fneur.2021.684791, 34335449 PMC8319239

[19] Wang Y Yang Y Wang Y Zhang J Zhai W Li S . Transcutaneous auricular vague nerve stimulation improved brain connection activity on patients of disorders of consciousness: a pilot study. J Tradit Chin Med. (2022) 42:463–71. doi: 10.19852/j.cnki.jtcm.2022.03.012 PMC9924658 35610018

[20] Hakon J Moghiseh M Poulsen I Øland CML Hansen CP Sabers A. Transcutaneous Vagus nerve stimulation in patients with severe traumatic brain injury: a feasibility trial. Neuromodulation. (2020) 23:859–64. doi: 10.1111/ner.13148, 32227429

[21] Noé E Ferri J Colomer C Moliner B O'Valle M Ugart P . Feasibility, safety and efficacy of transauricular vagus nerve stimulation in a cohort of patients with disorders of consciousness. Brain Stimul. (2020) 13:427–9. doi: 10.1016/j.brs.2019.12.005, 31866491

[22] Zhang Y Wang J Schnakers C He M Luo H Cheng L . Validation of the Chinese version of the coma recovery scale-revised (CRS-R). Brain Inj. (2019) 33:529–33. doi: 10.1080/02699052.2019.1566832, 30663434

[23] Giacino JT Kalmar K Whyte J. The JFK coma recovery scale-revised: measurement characteristics and diagnostic utility. Arch Phys Med Rehabil. (2004) 85:2020–9. doi: 10.1016/j.apmr.2004.02.033, 15605342

[24] Annen J Filippini MM Bonin E Cassol H Aubinet C Carrière M . Diagnostic accuracy of the CRS-R index in patients with disorders of consciousness. Brain Inj. (2019) 33:1409–12. doi: 10.1080/02699052.2019.1644376, 31319707

[25] Bodien YG Allanson J Cardone P Bonhomme A Carmona J Chatelle C . Cognitive motor dissociation in disorders of consciousness. N Engl J Med. (2024) 391:598–608. doi: 10.1056/NEJMoa2400645, 39141852 PMC7617195

[26] Bodien YG Vora I Barra A Chiang K Chatelle C Goostrey K . Feasibility and validity of the coma recovery scale-revised for accelerated standardized testing: a practical assessment tool for detecting consciousness in the intensive care unit. Ann Neurol. (2023) 94:919–24. doi: 10.1002/ana.26740, 37488068 PMC10701693

[27] Bodine CE Seu A Roth EA Wegener EE Magee WL. Examining the functionality of the MATADOC with the CRS-R: a pilot study. J Music Ther. (2020) 57:432–54. doi: 10.1093/jmt/thaa010, 32621608

[28] Zhou Y Sun Y He P Xiong Q Kang J Tang Y . The efficacy and safety of transcutaneous auricular vagus nerve stimulation for patients with minimally conscious state: a sham-controlled randomized double-blind clinical trial. Front Neurosci. (2023) 17:1323079. doi: 10.3389/fnins.2023.1323079, 38156271 PMC10752952

[29] Wannez S Heine L Thonnard M Gosseries O Laureys S Coma Science Group collaborators. The repetition of behavioral assessments in diagnosis of disorders of consciousness. Ann Neurol. (2017) 81:883–9. doi: 10.1002/ana.24962, 28543735

[30] Cortese MD Riganello F Arcuri F Pugliese ME Lucca LF Dolce G . Coma recovery scale-r: variability in the disorder of consciousness. BMC Neurol. (2015) 15:186. doi: 10.1186/s12883-015-0455-5, 26450569 PMC4599033

[31] Frangos E Ellrich J Komisaruk BR. Non-invasive access to the vagus nerve central projections via electrical stimulation of the external ear: fMRI evidence in humans. Brain Stimul. (2015) 8:624–36. doi: 10.1016/j.brs.2014.11.018, 25573069 PMC4458242

[32] Yakunina N Kim SS Nam E-C. Optimization of transcutaneous vagus nerve stimulation using functional MRI. Neuromodulation. (2017) 20:290–300. doi: 10.1111/ner.12541, 27898202

[33] Li Y Riganello F Yu J Vatrano M Shen M Cheng L . The autonomic response following taVNS predicts changes in level of consciousness in DoC patients. Sci Rep. (2025) 15:7317. doi: 10.1038/s41598-024-84029-4, 40025051 PMC11873156

[34] Katz DI Polyak M Coughlan D Nichols M Roche A. Natural history of recovery from brain injury after prolonged disorders of consciousness: outcome of patients admitted to inpatient rehabilitation with 1–4 year follow-up. Prog Brain Res. (2009) 177:73–88. doi: 10.1016/S0079-6123(09)17707-5, 19818896

[35] Estraneo A Moretta P Loreto V Lanzillo B Santoro L Trojano L. Late recovery after traumatic, anoxic, or hemorrhagic long-lasting vegetative state. Neurology. (2010) 75:239–45. doi: 10.1212/WNL.0b013e3181e8e8cc, 20554941

[36] Hróbjartsson A Thomsen ASS Emanuelsson F Tendal B Hilden J Boutron I . Observer bias in randomised clinical trials with binary outcomes: systematic review of trials with both blinded and non-blinded outcome assessors. BMJ. (2012) 344:e1119. doi: 10.1136/bmj.e1119, 22371859

[37] Moustgaard H Clayton GL Jones HE Boutron I Jørgensen L Laursen DRT . Impact of blinding on estimated treatment effects in randomised clinical trials: meta-epidemiological study. BMJ. (2020) 368:l6802. doi: 10.1136/bmj.l6802, 31964641 PMC7190062

[38] Button KS Ioannidis JPA Mokrysz C Nosek BA Flint J Robinson ESJ . Power failure: why small sample size undermines the reliability of neuroscience. Nat Rev Neurosci. (2013) 14:365–76. doi: 10.1038/nrn3475, 23571845

[39] Altman DG Bland JM. Interaction revisited: the difference between two estimates. BMJ. (2003) 326:219. doi: 10.1136/bmj.326.7382.219, 12543843 PMC1125071

[40] Li G Taljaard M Van den Heuvel ER Levine MAH Cook DJ Wells GA . An introduction to multiplicity issues in clinical trials: the what, why, when and how. Int J Epidemiol. (2017) 46:746–55. doi: 10.1093/ije/dyw320 28025257

[41] Bender R Lange S. Adjusting for multiple testing—when and how? J Clin Epidemiol. (2001) 54:343–9. doi: 10.1016/S0895-4356(00)00314-0, 11297884

[42] Whyte J Nakase-Richardson R Hammond FM McNamee S Giacino JT Kalmar K . Functional outcomes in traumatic disorders of consciousness: 5-year outcomes from the National Institute on Disability and Rehabilitation Research traumatic brain injury model systems. Arch Phys Med Rehabil. (2013) 94:1855–60. doi: 10.1016/j.apmr.2012.10.041, 23732164

[43] Hammond FM Giacino JT Nakase Richardson R Sherer M Zafonte RD Whyte J . Disorders of consciousness due to traumatic brain injury: functional status ten years post-injury. J Neurotrauma. (2019) 36:1136–46. doi: 10.1089/neu.2018.5954, 30226400

[44] Estraneo A Fiorenza S Magliacano A Formisano R Mattia D Grippo A . Multicenter prospective study on predictors of short-term outcome in disorders of consciousness. Neurology. (2020) 95:e1488–99. doi: 10.1212/WNL.0000000000010254, 32661102 PMC7713739

[45] Estraneo A de Bellis F Masotta O Loreto V Fiorenza S Lo Sapio M . Demographical and clinical indices for long-term evolution of patients in vegetative or in minimally conscious state. Brain Inj. (2019) 33:1633–9. doi: 10.1080/02699052.2019.1658220, 31533482

[46] Claassen J Doyle K Matory A Couch C Burger KM Velazquez A . Detection of brain activation in unresponsive patients with acute brain injury. N Engl J Med. (2019) 380:2497–505. doi: 10.1056/NEJMoa1812757, 31242361

[47] Sitt JD King JR El Karoui I Rohaut B Faugeras F Gramfort A . Large scale screening of neural signatures of consciousness in patients in a vegetative or minimally conscious state. Brain. (2014) 137:2258–70. doi: 10.1093/brain/awu141, 24919971 PMC4610185

